# Water-Blown Polyurethane Foams Showing a Reversible Shape-Memory Effect

**DOI:** 10.3390/polym8120412

**Published:** 2016-11-28

**Authors:** Elena Zharinova, Matthias Heuchel, Thomas Weigel, David Gerber, Karl Kratz, Andreas Lendlein

**Affiliations:** 1 Institute of Biomaterial Science, Helmholtz-Zentrum Geesthacht, Kantstr. 55, 14513 Teltow, Germany; elena.zharinova@hzg.de (E.Z.); matthias.heuchel@hzg.de (M.H.); thomas.weigel@hzg.de (T.W.); david.gerber@hzg.de (D.G.); karl.kratz@hzg.de (K.K.); 2 Institute of Chemistry and Biochemistry, Freie Universität Berlin, Takustrasse 3, 14195 Berlin, Germany

**Keywords:** polyurethane foams, reversible shape-memory effect, reversible actuation

## Abstract

Water-blown polyurethane (PU) foams are of enormous technological interest as they are widely applied in various fields, i.e., consumer goods, medicine, automotive or aerospace industries. The discovery of the one-way shape-memory effect in PU foams provided a fresh impetus for extensive investigations on porous polymeric actuators over the past decades. High expansion ratios during the shape-recovery are of special interest when big volume changes are required, for example to fill an aneurysm during micro-invasive surgery or save space during transportation. However, the need to program the foams before each operation cycle could be a drawback impeding the entry of shape-memory polymeric (SMP) foams to our daily life. Here, we showed that a reversible shape-memory effect (rSME) is achievable for polyurethane water-blown semicrystalline foams. We selected commercially available crystallizable poly(ε-caprolactone)-diols of different molecular weight for foams synthesis, followed by investigations of morphology, thermal, thermomechanical and shape-memory properties of obtained compositions. Densities of synthesized foams varied from 110 to 180 kg∙m^−3^, while peak melting temperatures were composition-dependent and changed from 36 to 47 °C, while the melting temperature interval was around 15 K. All semicrystalline foams exhibited excellent one-way SME with shape-fixity ratios slightly above 100% and shape-recovery ratios from the second cycle of 99%. The composition with broad distribution of molecular weights of poly(ε-caprolactone)-diols exhibited an rSME of about 12% upon cyclic heating and cooling from *T*_low_ = 10 °C and *T*_high_ = 47 °C. We anticipate that our experimental study opens a field of systematic investigation of rSMEs in porous polymeric materials on macro and micro scale and extend the application of water-blown polyurethane foams to, e.g., protective covers with zero thermal expansion or even cushions adjustable to a certain body shape.

## 1. Introduction

Polyurethane (PU) foams are widely spread in our daily life as a core part of the majority of comfort items, such as cushions or pillows. Other common applications include seals, packaging, shoe soiling and self-skinning articles. While the field of conventional PU foams has been extensively investigated and their large-scale manufacturing processes are well established, a completely new principle of foam’s operation, patented in the early 1990s [1], encouraged scientists for a new research aiming to integrate polyurethane foams into the field of smart materials. A series of studies describing polyurethane-based foams with shape-memory effect (SME) has been published [2,3,4], where, flexible when heated, the shape-memory polymer (SMP) foam can be shaped to a desired form, the introduced geometry can be stored during a required period and the recovery of the original shape of the SMP-foam can be triggered by regulating the ambient temperature. The shaping to a temporary form is commonly referred to as programming step. Conventionally, a SMP-foam is heated during programming to a *T*_prog_ until foam’s softening and compressed to a temporary shape. Keeping the degree of compression, the foam is cooled down to *T*_low_ so that the switching temperature *T*_sw_ (namely melting *T*_m_ or glass *T*_g_ transition temperature) is passed and the switching segments are “frozen” in a way that the programmed shape of the foam is kept. When the foam, in its temporary shape, is heated under load-free conditions above *T*_sw_, the original shape is recovered [5]. The process is repeatable; however the programming step requires an external force for each shape-memory cycle (so called one-way SME). Detailed quantification procedures of one-way SME in compression are described [5,6,7].

A large expansion in volume during the shape recovery is a target for most existing applications of SMP-foams. Thus, water-blown thermoplastic polyurethane foams, manufactured by Mitsubishi Heavy Industries, were investigated as a potential material for aerospace self-deployable elements [8,9]. Another example of highly expandable SME porous structures (the expansion ratio can be 10 times of the height of a compressed foam) are water-blown polyurethane foams based on low molecular weight triols and tetrols crosslinked with hexamethylene diisocyanate, which were tested as occlusion devices for aneurysms treatment [10,11]. Whereas in the latter example the single use of an SMP-foam is intended, the need to program an SMP-foam before every operation could deter their acquisition of new markets.

**Figure 1:**
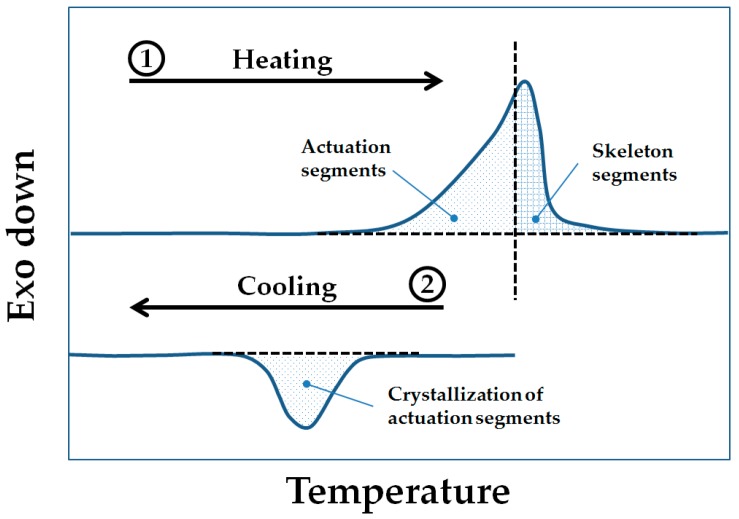
Calorimetric scheme of “separation” of actuation segments from skeleton segments of same polymer nature during heating (1) and subsequent crystallization of these actuation domains during cooling (2).

Therefore, we investigate here whether a reversible shape-change actuation (reversible SME or rSME) at ambient temperature is achievable for water-blown polyurethane foams. The currently reported polymeric rSME materials have actuation domains and skeleton domains of same or different chemical composition [12,13]. The actuation domains contract upon heating and elongate during crystallization, whereas skeleton structural domains remain unchanged during a temperature shape-memory cycle and retain the overall shape [14]. Given that the directed crystallization causes rSME, we aimed to prepare semicrystalline polymeric foams having a melting transition region broad enough to accommodate both actuation and skeleton domains (Figure 1). Under this concept, *T*_high_ is adjusted during the rSME cycle to trigger the melting of only a part of the crystalline phase of the foam.

The architecture and the direction of stress, in addition to the composition, determine a mechanical response of the foam to the externally applied force and, as a result, the SME. Hence, it was shown, that hierarchically structured foams obtained via thermally-induced phase separation exhibited improved shape recovery values compared to foams with homogeneous structure [15]. Despite the importance of microstructure only few studies of SMP-foams focused their research on a single pore level, whereas the majority of studies cover one-way SME on the level of macroscopic geometry changes. In the current research, we therefore show the results of investigations of one-way and rSME on both, micro- and macrolevels, for programming regimes in compression, tension and bending modes.

**Figure 2:**
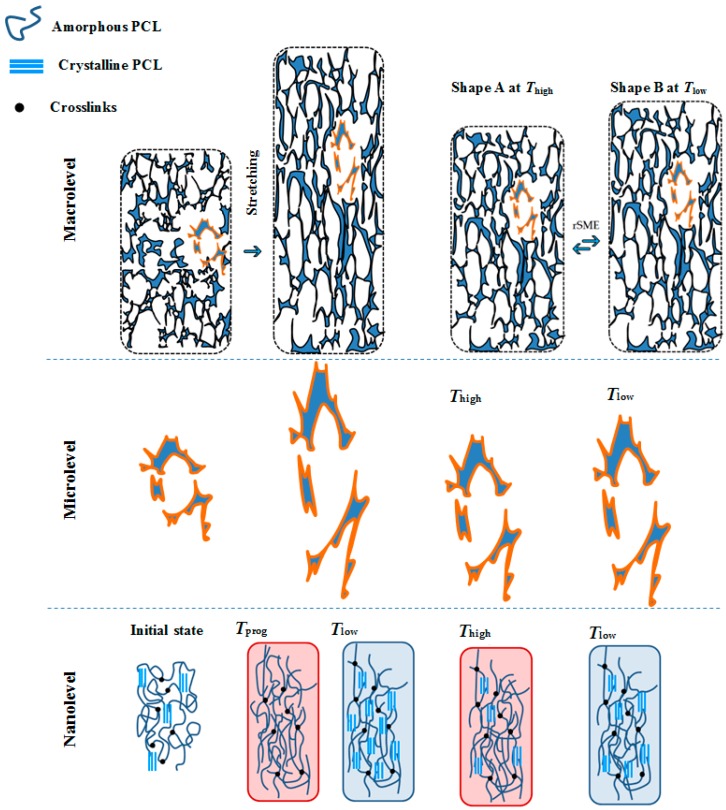
Scheme of performance of a semicrystalline foam during programming and a reversible shape-memory effect (rSME) cycle on the three different structural levels. From left to right: a foam sample is programmed at *T*_prog_ in tension mode; a foam is contracted upon melting of actuation domains at *T*_high_ (Shape A) and elongated upon crystallization of actuation domains at *T*_low_ (Shape B). PCL: poly(ε-caprolactone).

For rSME, the programming step aims to induce the orientation of macromolecules and therefore to give a direction to actuation and skeleton domains upon cooling of the material. Since in foams there is no preferred direction of single elements (pores’ walls and struts) upon uniaxial compression [16], we hypothesize, in addition, that programming efficiency in tension mode could be higher than in the conventional compression mode. When the foam is stretched at *T*_prog_, the most structural elements should be elongated in the direction of stretching, and thereby the orientation of actuation and skeleton domains at *T*_low_ should be more pronounced than in compression mode (see Figure 2). Thus, the melting of actuation segments at *T*_high_ will cause a shrinkage of pores and a decrease of the length of the sample on macrolevel (see Figure 2, shape A *T*_high_). The crystallization of actuation segments on nanolevel will cause elongation of pores upon cooling and a corresponding elongation of the sample on the macrolevel (see Figure 2, shape B at *T*_low_).

Systems having a reversible SME often contain poly(ε-caprolactone) (PCL) switching and actuator skeleton domains [14,17]. In addition, polycaprolactone-based systems are considered to be biodegradable and biocompatible. Hence, it was decided to use commercially available crystallizable linear poly(ε-caprolactone)-diols (PCL-diols) of different molecular weights as a polyester-polyol type precursor to introduce both switching and skeleton segments. Diphenylmethane diisocyanate (MDI) was chosen as second component of the polyurethane reaction. It is well understood that the shape recovery range of foams with SME is related to the crosslink density; even the very low density foams with high crosslink density exhibit good shape-memory properties [14,15]. Therefore, the MDI weight ratio was kept high to achieve high yield of linear PCL-diols incorporation into the foams’ network.

The article presents the comprehensive investigations of morphology, thermomechanical and shape-memory properties of obtained water-blown PU foams based on PCL-diols. At first, gel content and foam density were determined for all specimens obtained. In parallel, micro-computed tomography (µCT) was used to investigate the morphology of the studied foams. Thermal properties were studied using differential scanning calorimetry (DSC). Dynamic mechanical thermal analysis (DMTA) was carried out to examine thermo-mechanical properties of compositions obtained. We evaluated one-way SME in compression mode and reversible shape-memory properties in compression and tension load-free modes using the thermomechanical cyclic experiments. In addition, we explored shape-memory properties on pores level of chosen foams using scanning electron microscopy (SEM) and micro-computed tomography. Finally, we demonstrated the rSME of a water-blown polyurethane foam programmed in bending mode.

## 2. Materials and Methods

### 2.1. Materials

Poly(*ε*-caprolactone) diols (PCL-diols) with different molecular weights (2000, 4000 and 8000 g∙mol^−1^) were purchased from Solvay (Warrington, UK); properties of the PCL-diols are summarized in Table 1. The 4,4′-diphenylmethan-diisocyanat (MDI) was ordered from Alfa Aesar (Karlsruhe, Germany). The chain extender and crosslinker diethanol amine (DEOA) was purchased from Acros Organics (Geel, Belgium). As balanced amine catalyst Dabco^®^ BLV was used, which represents a 3:1 blend of gelling and blowing catalysts (3:1 blend of triethylenediamine and 2-(dimethylamino)ethyl ether). Stannous octoate (Dabco^®^ T-9) was used to support polyol-isocyanate reaction. Both catalysts were kindly provided by Air Products and Chemicals, Inc. (Allentown, PA, USA). All materials were used as received. Deionized water (conductivity of 1 µS∙cm^−1^) was used as chemical blowing agent.

### 2.2. Foam Synthesis

In order to study the influence of crystallizable polyol on thermal properties of crosslinked foams and to achieve a melting transition range acceptable for rSME actuation, PCL-diols of three molecular weights were used: PCL-diol of around 2000 g∙mol^−1^ (2k PCL-diol), PCL-diol of around 4000 g∙mol^−1^ (4k PCL-diol), and PCL-diol of around 8000 g∙mol^−1^ (8k PCL-diol) and their combinations. Compositions of the foams are given in Table 2. In the composition code cPCL-X/Y/Zk-123, cPCL denotes crosslinked PCL, X/Y/Zk stands for number average molecular weight of PCL-diol used, and the last three digits represent the density of the obtained foams (see Table 3).

To fabricate our foam samples, standard two-step foaming technique was used. Lab-scale 300 mL polypropylene (PP) beakers served as molding forms for foam free-rise and post-curing. At first, a polyol (or a mixture of polyols) was melted in an oil bath heated to 70 °C and rigorously blended during 2 min with catalysts, blowing agent (water) and chain extender; secondly, MDI (preliminary melted in an oven at 70 °C) was added to the mixture and compositions were additionally stirred for 20 s (or until the beginning of the foaming). A high-speed 200 W/12,000 rpm. mixer with angled disk stirrer (Gastro 200, Unold AG, Hockenheim, Germany) was used for both mixing steps.

The freshly prepared foams inside the beakers were placed into an oven at 70 °C and kept there for one week for post-curing.

### 2.3. Preparation of Samples

To obtain test specimens, the manufactured foam samples were frozen with liquid nitrogen and then cut with a micro-band saw (MBS 240/E, Proxxon GmbH, Trier, Germany) into 10 mm high slices in the directions parallel and perpendicular to the foam rise. For thermomechanical and shape-memory cyclic tests (performed in compression mode) as well as for the density measurements, cylindrical samples (diameter: 20 mm; height: 10 mm) were punched out with a hand press (Schmidt Technology Corp., Cranberry, PA, USA) using a round-type stencil.

For the shape-memory tests in tension mode, the slices were cut to bars of 3 mm × 10 mm × 30 mm size (parallel and perpendicular to foam rise).

### 2.4. Characterization of Crosslinked Poly(*ε*-caprolactone) (cPCL)-Based Foams

#### 2.4.1. Evaluation of Composition and Morphology of cPCL-Based Foams

To evaluate the level of crosslinking (gel content), extraction experiments in *N*,*N*-dimethylformamide (DMF) solvent (Sigma-Aldrich Co., St. Louis, MO, USA) were carried out. Small samples were cut off the foam (initial sample mass *m_iso_* < 0.15 g), weighted and immersed into DMF for 48 h. Swollen samples were taken out from the solvent, and excess solvent was carefully blotted with cotton towels. Afterwards, the samples were dried during 48 h at 60 °C under vacuum (the constant weight was controlled). The weight of the dry samples *m_dry_* was measured and the gel fraction (*G*) was calculated using Equation (1):

(1) $$G=\;\frac{m_{dry}}{m_{iso}}\times 100\%$$

Five measurements were performed for each composition to assess the standard deviation.

The apparent core density was calculated from the weight and volume of the prepared cylindrical specimens. The volume was determined from the dimensions of cylindrical foam specimens. Height and diameter of the foam cylinders were measured with a digital caliper (Garant ABS, Hoffmann GmbH, Achim, Germany). Each specimen was weighted using a laboratory balance (Sartorius ME254S, Sartorius AG, Goettingen, Germany). For each formulation type a minimum of 5 specimens were measured.

The cellular structures, pores size, volume pore size distribution and pores interconnectivity were evaluated using a micro-computed tomography (µCT) instrument (Procon X-ray GmbH, Sarstedt, Germany) with X-ray source L9181-02 (Hamamatsu Photonics K.K., Hamamatsu, Japan) and detector C7942SK-05 (Hamamatsu Photonics K.K., Hamamatsu, Japan). Accelerating voltage and current for X-ray source tube were set as 40 kV and 0.2 mA, respectively. The reconstruction of foam elements and calculations were done with MAVI Software (Version 1.4, Fraunhofer ITWM, Kaiserslautern, Germany).

To estimate the content of closed pores in a µCT experiment, a foam sample was immersed into oil for 48 h. The oil penetrated through interconnected pores while the closed pores kept still air inside. Therefore, on the µCT image of the sample immersed into oil, only closed pores were counted. The volume ratio of closed cells content φ*_cc_* was estimated using Equation (2),

(2) $$\phi_{cc}=\frac{\phi_{vc}}{\phi_{v}}\times 100\%$$

where φ*_vc_* is a volume fraction of closed cells and φ*_v_* is total volume fraction of found cells.

#### 2.4.2. Thermomechanical Characterization

Thermal properties were evaluated using a DSC 204 calorimeter (Netzsch, Selb, Germany). Heating and cooling rates were 3 K∙min^−1^; the scanning range was from −80 to 100 °C. Areas under the melting and crystallization peaks were used to calculate the corresponding enthalpies *ΔH*_m_ (*T_m_*). Weight percent crystallinity (*DOC*) was calculated according to Equation (3) [18],

(3) $$DOC=\frac{\Delta H_{m\;}\left(T_{m}\right)}{\Delta H_{m}^{0}\left(T_{m}^{0}\right)}\times 100\%$$

where $\Delta H_{m}^{0}\left(T_{m}^{0}\right)$ is the enthalpy of melting of 100% crystalline polycaprolactone. We used a literature value of 139.3 J∙g^−1^ [18].

Thermomechanical properties were assessed through DMTA compressive studies on a GABO Eplexor 25 N (Testanlagen GmbH, Ahlden, Germany) on cylindrical specimens (all perpendicular to free foam rise) at 5% dynamic load strain and 10% static load strain with a frequency of 10 Hz. The temperature ranged from −80 to 70 °C and the heating rate was 2 K∙min^−1^. The contact force was set to 0.05 N.

#### 2.4.3. Thermomechanical Tests in Compression Mode, Cyclic Thermomechanical Tests in Compression and Tensile Modes

The compression tests and the quantification of SME were carried-out using a Zwick universal test machine (Zwick GmbH, Ulm, Germany) equipped with a thermo-chamber and temperature controller (Eurotherm Regler, Limburg, Germany).

*One-way shape-memory cycles* consisted of a programming step where the specimens were compressed at *T*_prog_ = 60 °C to *ε*_prog_ = 60% of their initial height. The compression rate was 5 mm∙min^−1^. While keeping the degree of compression, samples then were cooled to *T*_low_ = 10 °C and equilibrated at *T*_low_ for 10 min. The load was released afterwards and the sample was heated to *T*_prog_ = 60 °C while the recovery of the height was recorded. The recovery was followed by equilibration at *T*_prog_ for 10 min. The speed of force release after equilibration at *T*_low_ was 0.9 N∙mm^−1^; a contact force was 0.5 N. Heating and cooling rates were set to 5 K∙min^−1^. The cycle was repeated three times for each measurement. The fixity and recovery ratios were calculated according to the procedure described elsewhere [19].

*Reversible SME tests in compression mode.* For programming, a cylindrical specimen of cPCL-2/4/8k-180 was compressed at *T*_prog_ = 60 °C to *ε*_prog_ = 60% of its initial height with the compression rate of 5 mm∙min^−1^. Then the specimen was cooled to *T*_low_ = 13 °C with 3 or 1 K∙min^−1^. After 10 min of equilibration, the load was released and the sample was repeatedly heated and cooled from to *T*_low_ = 13 °C to *T*_high_ = 51 °C in a load-free regime. Heating and cooling rates were 1 or 3 K∙min^−1^. The speed of force release after equilibration at *T*_low_ was 0.9 N∙mm^−1^; the contact force was 0.2 N. Taking into account that the direction of rSME in compression mode is opposite to the rSME in tension mode, we modified the equation to calculate the reversible strain ε_rev_ proposed elsewhere [14] as follows,

(4) $$\varepsilon_{rev}=-\frac{L_{low}-L_{high}}{L_{low}}\;\times 100\%$$

where *L*_low_ is the height of the specimen at *T*_low_ and *L*_high_ is the height of the specimen at *T*_high_.

*Reversible SME tests in tension mode.* Bars (cut parallel or perpendicular to foam rise) were stretched to 60% of its original length at *T*_prog_ = 60 °C with the stretching rate of 5 mm∙min^−1^. Then the specimen was cooled to *T*_low_ (was varied) with 3 K∙min^−1^. After 10 min of equilibration the load was released and the sample was repeatedly heated and cooled from to *T*_low_ (was varied) to *T*_high_ (was varied) in a load-free regime. The speed of force release after equilibration at *T*_low_ was 0.9 N∙mm^−1^; a contact force was 0.2 N. The values of the reversible strains *ε*_rev_ obtained were calculated as it was described elsewhere [14].

*Reversible SME tests in bending mode.* A beam-shaped specimen with approximately 30 mm × 30 mm at its base and around 5 mm of height was cut off the foam. The large specimen was used to demonstrate the performance of the programmed polymeric foam upon cyclic heating and cooling. The obtained sample was heated to *T*_prog_ = 60 °C and bended along its large side perpendicular to the foam rise direction. The programmed specimen was cut from one side and the contours were marked with a black marker to assure a clear tracking of the geometry change. The tests were performed inside of a transparent box. The heating and cooling were performed with the electronic heat gun Steinel HL 2010 E (Steinel America Inc., Bloomington, MN, USA) and the nitrogen air stream supplied through a vessel filled with liquid nitrogen, respectively. The temperature was controlled with thermocouple thermometer Digi-Sense Dual J-T-E-K (Cole-Parmer Instrument Co., Vernon Hills, IL, USA). The video record was performed from the front view, while the photos were done at *T*_low_ and *T*_high_ for every temperature cycle with photo camera from the side keeping the same position and photo angle. Change of an angle between the bend sides at *T*_low_ and *T*_high_ from the side view was analyzed from the photos using the ImageJ software (version 1.5.1.; National Institutes of Health, Bethesda, MD, USA).

### 2.5. Characterization of Shape-Memory Effect (SME) on Microlevel

The investigations of SME on microlevel were done in-situ using µCT on a sample holder equipped with a plastic cover of low X-ray absorption. Temperature of the sample holder was controlled with Peltier element equipped with water cooling system; the accuracy was of ±1 °C. The heating, cooling, and equilibration were done directly inside the µCT chamber.

One-way in-situ SME tests were done in compression mode. The programming was done directly on the sample holder inside the µCT chamber. For this, the foam specimen of the cylindrical sample of 8 mm in diameter and 10 mm height was cut out from the foam, heated to *T*_prog_ = 60 °C and compressed to *ε*_prog_ = 60%. While the compression was maintained, the specimen was cooled to 20 °C (compressed state). The foam then was heated to 60 °C to trigger the recovery. Measurements were performed for all three states (initial, compressed and recovered).

For reversible SME tests, bar-shaped foam specimens were preliminary programmed by tension at *T*_prog_ = 60 °C to *ε*_prog_ = 60% using the Zwick universal test machine. Then the sample was placed on the sample holder inside µCT chamber, heated to *T*_high_ = 49 °C, equilibrated for at least 30 min, cooled to *T*_low_ = 22 °C and equilibrated again for at least 30 min. Measurements were performed at *T*_high_ and *T*_low_.

Additionally, a one-way recovery study from the compressed state was done with a SEM Phenom G2 Pro (Phenom-World BV, Eindhoven, The Netherlands). The foam specimen with a thickness of about 2 mm was placed between the plates of a microscrew device in a way that the whole place between plates was occupied by the foam. The initial distance between the plates microscrew device was measured with a digital caliper. The sample inside the device was placed into the oven heated to *T*_prog_ = 60 °C, equilibrated for around 30 min and compressed to a compression ratio of around 80%. After cooling to room temperature, the distance between plates of microscrew device was measured again and the compression ratio was calculated from the two values obtained. The compressed specimen was placed inside the microscrew device into a sample holder of SEM for investigation of the microstructure. For the investigation of each further recovery step, the sample was heated outside of SEM at *T*_prog_ = 60 °C and the screw was released for one turn, the specimen was equilibrated at *T*_prog_ and cooled to room temperature. The procedure was repeated until the initial position of the screw was reached, which corresponds to a full recovery of a foam specimen.

## 3. Results and Discussion

A series of water-blown polyurethane foams was prepared from linear PCL-diols. In order to obtain a range of densities and thermal and thermomechanical properties different number average molecular weights and their combinations were used without any change of the processing conditions.

### 3.1. Foam Composition and Morphology

Density and gel content of the obtained foams vary significantly with change of low molecular weight polyol content (Table 3). Presence of the PCL-diol with 2000 g∙mol^−1^ in the compositions gives decreased density values (for cPCL-2k-110 and cPCL-2/4k-130) and an increase in gel content compared to compositions with higher molecular weight PCL-diols (cPCL-2/8k-170, cPCL-2/4/8k-180 and cPCL-4k-220).

Based on obtained values of densities, we here establish an internal classification of foams: foams with *ρ* < 150 kg∙m^−3^ were considered as low density foams, while foams with *ρ* > 200 kg∙m^−3^ are considered as high density foams. The density values in-between these ranges can be treated as an intermediate range of densities.

When the polyols with higher molecular weight (PCL 4000 g∙mol^−1^ and PCL 8000 g∙mol^−1^) are introduced, reduced gel content and density growth should be related to increased viscosity of those formulations, which could hinder crosslinking, homogenization of reacting mixture and reduce the amount of bubbles introduced during mixing.

**Figure 3:**
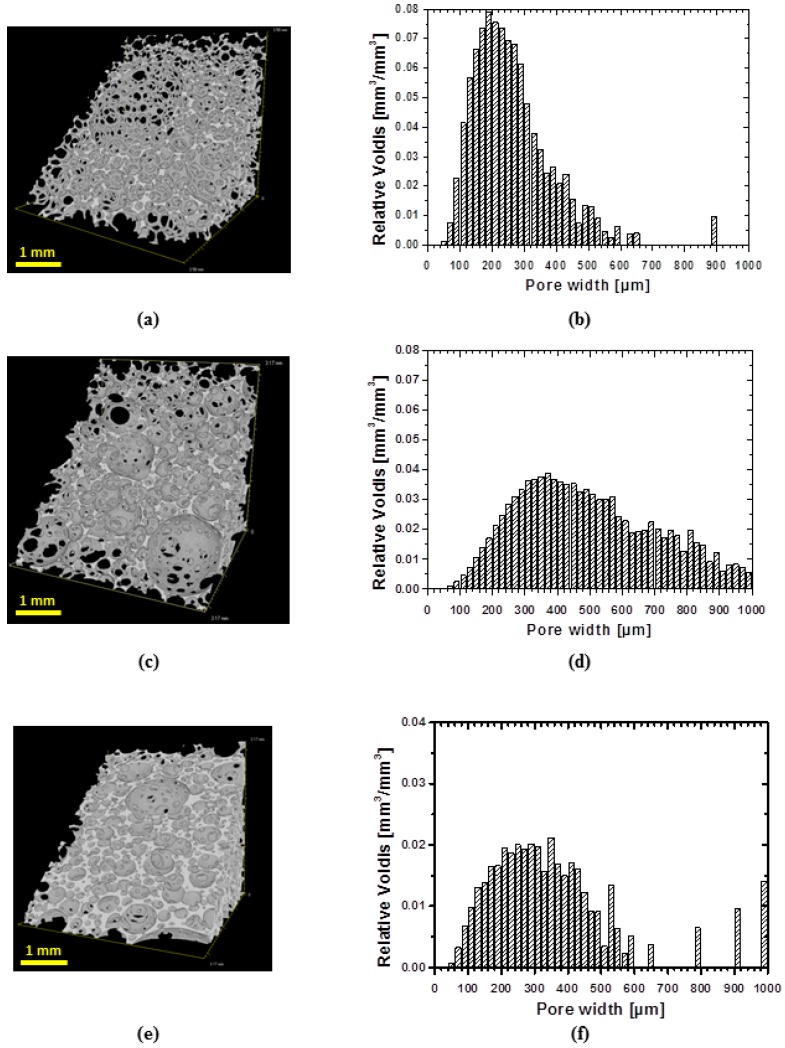
µCT images of 3D structure of water-blown PCL-polyol based Polyurethane (PU) foams illustrating the effect of density on foam architecture: (**a**) cPCL-2k-110 with porosity = 0.88 ± 0.09; (**c**) cPCL-2/4/8k-180 with porosity = 0.85 ± 0.09; and (**e**) cPCL-4k-220 with porosity = 0.73 ± 0.07. Pores volume distribution in relation to the pore sizes are depicted for: (**b**) cPCL-2k-110; (**d**) cPCL-2/4/8k-180; and (**f**) cPCL-4k-220.

In order to evaluate the architecture of obtained foams, a three-dimensional analysis of structure was performed using µCT. All samples exhibit shell-like structure with windows (Figure 3a,c,e). However, low density samples show more developed windows (Figure 3a) than specimens of higher density (Figure 3c). The specimen of highest density exhibits the less developed windows in the row of manufactured foams (Figure 3e). The dependence of cell structure on the foam’s density is in good agreement with the existing literature [20].

µCT technique allows also evaluating a pore size distribution (Figure 3b,d,f). Generally, for all specimens the pore size distribution is broad. Thus, around 70% of volume of specimen cPCL-2k-110 is occupied by pores of widths within 100–300 µm range, while the highest abundance of pores by volume was occupied by pores of the size of 200 µm (Figure 3b). The sample cPCL-2/4/8k-180 with the intermediate density possesses a mean pore size of around 190 ± 30 µm (by size) with the major part of pores located between 200 and 600 µm (by volume) (see Figure 3d).

For specimen cPCL-4k-220, 70% of volume is occupied with pores sized between 150 and 450 µm, where the majority of the pores by volume were located between 200 and 300 µm (Figure 3f).

**Figure 4:**
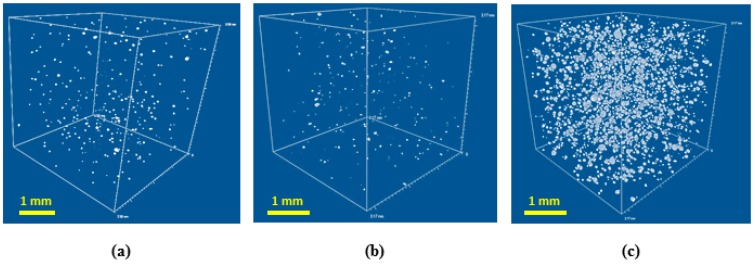
µCT image of closed pores included into foam volume for: (**a**) cPCL-2k-110, volume fraction of closed cells ≈ 0.05%; (**b**) cPCL-2/4/8k-180, volume fraction of closed cells ≈ 0.04%; and (**c**) cPCL-4k-220, volume fraction of closed cells ≈ 1.7%.

In general, the relatively large pore size for all formulations could be explained by cell coalescence and coarsing during growing foam bubbles in absence of surfactant [21]. The ratio of closed to open pores was estimated using µCT (see Figure 4).

Fraction of closed cells is increasing for the high density foams. However, the maximum volume fraction of closed cells obtained is negligible, so the architecture of pores can be considered as interconnected for all specimens studied.

### 3.2. Thermal Properties of Semicrystalline Polyurethane (PU)-Foams

For better SME good crystallization behavior is necessary. DSC experiments were performed to assess crystallization behavior of obtained foams.

**Figure 5:**
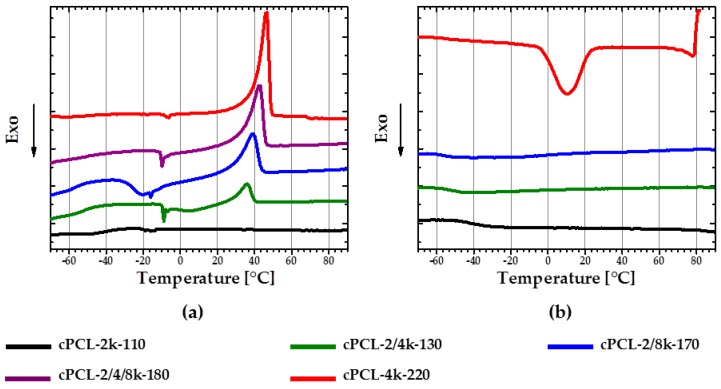
(**a**) Differential scanning calorimetry (DSC) second heating; and (**b**) DSC first cooling curves of polyurethane foams with different polyol chain lengths. Heating and cooling rates were 3 K∙min^−1^.

With a decrease of the content of 2k PCL-diol in the foam composition, corresponding PCL melting peak appears to be more pronounced during second heating. In addition, the melting temperatures are shifted to lower values with an increase of the short 2k PCL-diol content (Figure 5a). Thus, cPCL-4k-220 has the biggest and sharpest melting peak with *T*_m_ = 45 ± 2 °C, while the melting peak of cPCL-2/4k-130 is poorly pronounced and has a *T*_m_ = 36 ± 1.5 °C. The foam prepared solely from the short PCL-diols exhibits an amorphous behavior. At the same time, all of the semicrystalline formulations exhibit a cold crystallization during heating (see Figure 5a). Tendency to cold crystallization becomes more distinct with an increase of content of the longest 8k PCL-diols in composition.

The pronounced crystallization during cooling was only observed for two formulations: cPCL-4k-220 with a *T*_c_ peak at around 10 °C and cPCL-2/4/8k-180 with a peak at around −14 °C.

Generally, three variables influencing the crystallization process were observed: The foam density, the yield of the crosslinking reaction, and the molecular weights of the polyols used. With the increase of foam density, the crystallization and consequently the melting are obviously more pronounced on DSC curves (see Figure 5a) (the density is increasing in the row from cPCL-2/4k-130 via cPCL-2/8k-170 to cPCL-4k-220). However, density alone does not fully determine the structure. Indeed, the foams with similar density (cPCL-2/8k-170 and cPCL-2/4/8k-180) exhibit different calorimetric properties: the cPCL-2/8k-170 does not exhibit crystallization during cooling but shows broad cold crystallization (Figure 5a, blue line). For the foam cPCL-2/4/8k-180, the cold crystallization is poorly expressed, although its crystallization is clearly visible during cooling in a range from 10 to −20 °C (Figure 5b, purple line). Absence of crystallization during cooling and presence of cold crystallization for cPCL-2/8k-170 could be related to higher immobility of PCL chains compared to the cPCL-2/4/8k-180. Hindrance of chain mobility could be related to the increase of crosslinking yield for cPCL-2/8k-170 (*G* = 89% ± 6%) compared to cPCL-2/4/8k-180 (*G* = 86% ± 2%). The shift of melting and crystallization temperatures to lower values should be related to the decrease in the mobility of PCL chains with the increase of the gel content.

**Figure 6:**
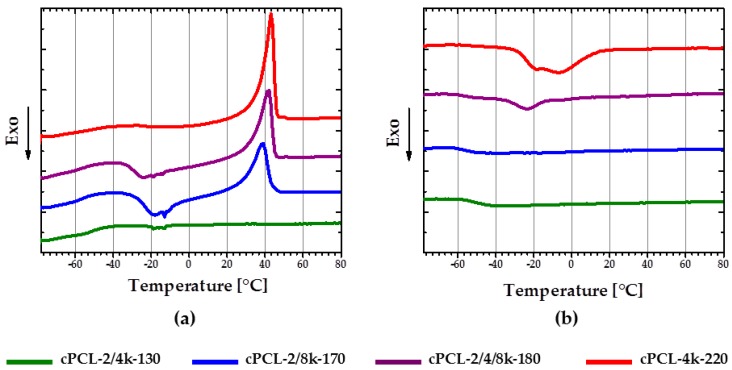
(**a**) DSC second heating; and (**b**) DSC first cooling curves of polyurethane foams with different polyol chain lengths after extraction in *N*,*N*-dimethylformamide (DMF). Heating and cooling rates were 3 K∙min^−1^.

To exclude the influence of the chains not incorporated to the foam network, the calorimetric measurements were also performed for foams extracted in DMF solvent (see Figure 6). Specimen cPCL-2/4/8k-180 (Figure 6a, purple curve) exhibits similar behavior to cPCL-2/8k-170 (Figure 6a, blue curve) during DSC experiment after extraction. The crystallization peak on the cooling curve of cPCL-2/4/8k-180 becomes tiny, and the cold crystallization was more pronounced in comparison to the untreated sample. Meanwhile, the heating and cooling curves for cPCL-2/8k-170 did not change significantly after the extraction procedure.

A melting peak of the low density specimen cPCL-2/4k-130 is absent on the second heating curve (Figure 6a, green curve) after extraction. Absence of melting peaks after extraction could be related to the fact that all free chains of PCL-diol, which could support the crystallization, were washed out by the solvent. The pronounced crystallization peak for the foam with PCL-diol of 4000 g∙mol^−1^ together with small crystallization peak of cPCL-2/4/8k-180 after extraction could be related to some of the polyol chains being incorporated to the system only on one side (“grafted”) and did not lose their mobility.

### 3.3. Thermomechanical Properties

**Figure 7:**
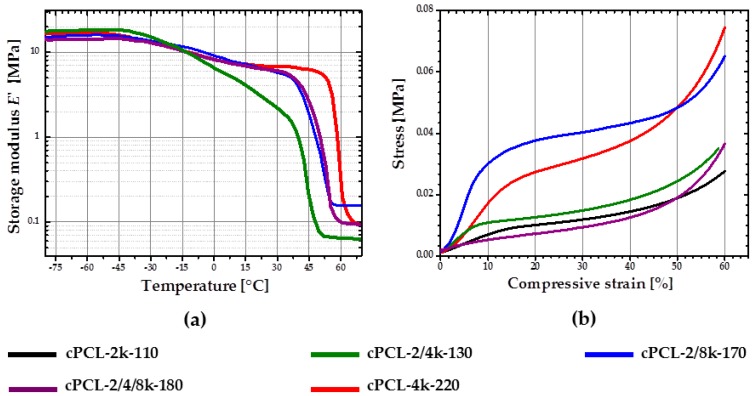
(**a**) Storage modulus dependence on the temperature for semicrystalline foams with different degree of crystallinity before extraction; and (**b**) stress–strain dependence at 60 °C for polyurethane foams with different polyol chain length (compression speed was 5 mm∙min^−1^).

DMTA measurements were performed to obtain information on thermomechanical properties of the foams. Figure 7a depicts experimental DMTA results for semicrystalline foams with different degree of crystallinity. Due to the lack of crystalline phase, which is a prerequisite for an rSME, the cPCL-2k-110 is not discussed here. At temperatures below *T*_g_ (*T*_g_ varies from around −40 °C for cPCL-2/4k-130 to around −60°C for cPCL-4k-220), all foams behave similarly. With temperature increase the storage modulus shows a conventional decay in two steps at *T*_g_ and *T*_m_ (Figure 7a). In addition, higher difference between *E*-modulus at *T*_g_ and *T*_m_ results in steeper decay of *E*-modulus on the interval between *T*_g_ and *T*_m_. *T*_m_ obtained with DMTA varies from around 40 °C for cPCL-2/4k-130 to around 55 °C for cPCL-4k-220. The *E*-modulus at *T*_m_ is around 5 MPa for formulations with high and intermediate densities and around 1 MPa for cPCL-2/4k-130. The storage modulus exhibits plateau at temperatures above *T*_m_ for all formulations studied.

The shift of *T*_m_ to lower temperatures in the array from high to low density samples is suggested to be related to the reduced degree of crystallinity for foams with lower densities. In addition, the higher the amount of amorphous phase is in a foam (e.g., for the cPCL-2/4k-130), the more pronounced is the *E*-modulus-temperature dependence on the interval between *T*_g_ and *T*_m_. The values of *E*-modulus at *T*_m_ are probably mainly related to the combined influence of foam density and degree of crystallinity. By changing *DOC* of foams of a given density through composition or simply by temperature change, their *E*-modulus and correspondingly their hardness can be adjusted.

The plateau values of *E*-modulus at temperatures above 60 °C for different formulations are influenced by the crosslink density and densities of specimens. Taking into account the variation in densities for different specimens, the crosslink density and the amount of entanglements can be assessed from the plateau values of *E*-modulus at temperatures above 60 °C for specimens tested after extraction in DMF. The highest crosslink density could be assigned for the foam cPCL-2/8k-170 (see Figure S1 and Table S1 in the supporting information [22]); the lowest crosslink density is found for cPCL-4k-220. These data are in correlation with conclusions drawn about chain mobilities based on DSC experiments for high-density foam.

Figure 7b depicts stress–strain diagrams for the foams made in compression mode above the melting intervals for all specimens. The stress–strain curves for all samples exhibit classical behavior for open-cells foams with three clearly distinguishable regimes: Linear, plateau and densification [16,20]. However, the different profiles of the foams are likely to be related to the combined contribution of foam´s architecture and composition of the particular specimen.

Based on the results of the characterizations described above the intermediate density foams (cPCL-2/8k-170 and cPCL-2/4/8k-180) were chosen for SME characterizations due to their relatively high crystallinity, acceptable crystallization behavior and gel content. The foams were used without additional treatment with DMF. 

### 3.4. Shape-Memory Properties

SME properties were investigated on two scales. On the macroscale, the typical heating–cooling experiments were performed with the foam samples. Differently to the bulk materials, the SME behavior of foams is accompanied with changes in the microarchitecture of the foams. Whereas the SME effect on macro level is represented by general change of foam’s shape, the corresponding effects on micro level are accompanied by the deformation of the pores.

We believe that for the porous structures the structural changes on micro level should mainly influence the programming efficiency of the foams, hence the nanostructure. The SME effects on microscale were investigated with µCT and SEM.

#### 3.4.1. One-Way SME on Macroscale

**Figure 8:**
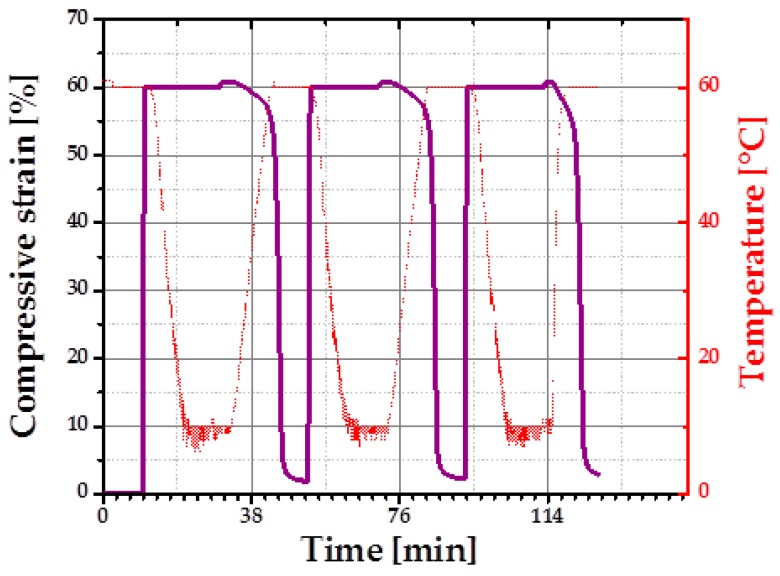
One-way SME for cPCL-2/4/8k-180 (heating and cooling rates were 5 K∙min^−1^).

For quantification of one-way SME of an intermediate density specimen (cPCL-2/4/8k-180) the thermo-mechanical cyclic tests in compression mode were performed using a universal testing machine. Figure 8 depicts three programming and stress-free recovery cycles of the chosen specimen. Semicrystalline cPCL-2/4/8k-180 exhibits good shape fixity at *T*_low_ (shape fixity was 101% ± 0.2%). Recovery ratio was 98% ± 0.5% for the first cycle (training cycle) and 99.5% ± 0.5% for the following second and third cycles.

A fixity ratio above 100% was reported previously for polymeric SME foams containing crystalline PCL domains. Such behavior was attributed to the compression-induced recrystallization [23]. Almost 100% of shape recovery ratios are related to satisfactory crosslink-density assuring shape stability of the specimen at *T*_prog_. The recovery process of the cPCL-2/4/8k-180 is shown in the supplementary Information (Figure S2).

#### 3.4.2. One-Way SME on Microscale

**Figure 9:**
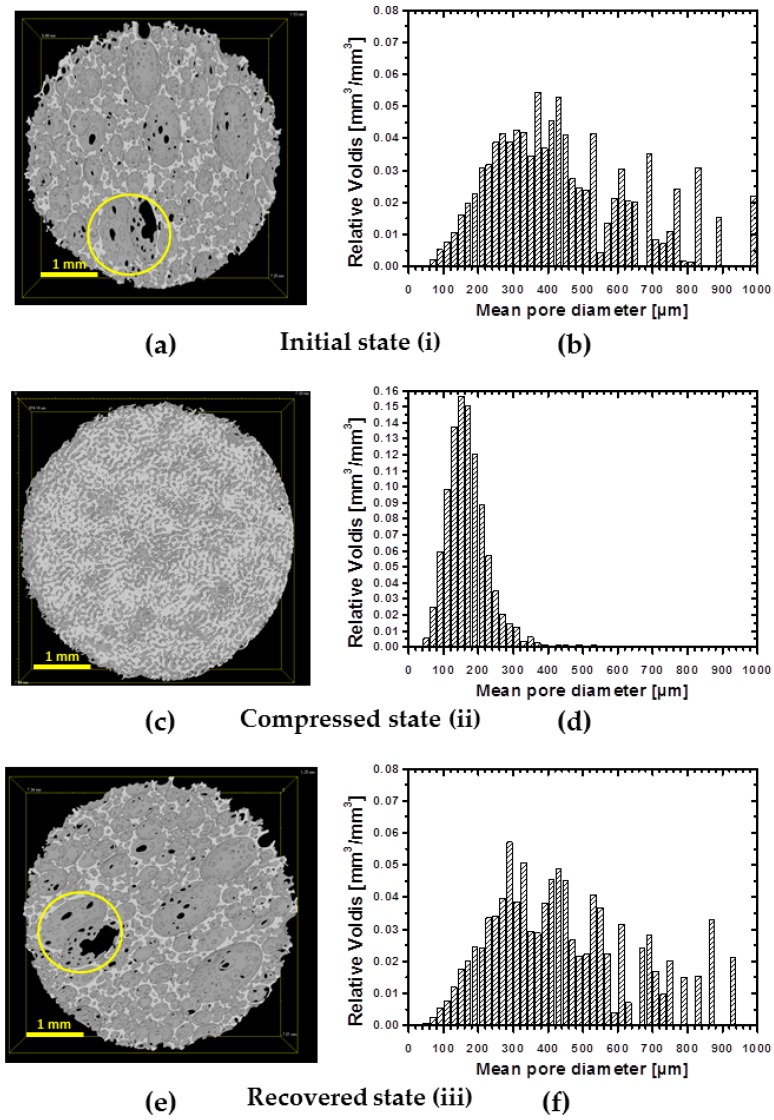
Three-dimensional imaging of initial state of the cPCL-2/8k-170 specimen (**a**); specimen compressed in situ to 60% cPCL-2/8k-170 (**c**); and recovered specimen cPCL-2/8k-170 (**e**). The respective pores volume distribution are given for: initial (**b**); compressed (**d**); and recovered (**f**) states. Yellow circles indicate the same site in the foam in its initial and recovered states.

At first, a one-way shape-memory performance of foam’s pores was evaluated with µCT. Figure 9a,c,e depicts µCT images of: initial (i), compressed to 60% (ii) and recovered (iii) states of the specimen. To ensure that the same site is observed on the image, a cross-section of 1 mm thickness from the bottom of the specimen was taken for the view. Indeed, the same spot in the specimen before compression and after recovery are detectable (Figure 9a,e marked with yellow circle). No substantial changes are observed after the recovery of the foam. The compressed state is presented in Figure 9c. Visually, the size of voids is decreased significantly and the structure became dense. Pores volume distributions are depicted in Figure 9b,d,f for each deformation state. There is a strong shift of the pore volumes distribution to smaller size values for the specimen in a compressed state (from around 400 µm by volume at initial state to 200 µm by volume at the compressed state). Such volume decrease would confirm the buckled state of the cell walls taking into account that, due to the limited resolution of µCT, when the cell walls approach to each other, the software would count the space formed as a separate object during reconstruction.

Therefore, in a buckled state, the number of detected objects would be increased, however they should be of a small size range. Indeed, the pore number increased by one order of magnitude in a compressed state and was around 420 objects per mm^3^ compared to the undeformed state with only 53 objects per mm^3^. The pore size distribution after recovery remains similar to the original state of the foam. Large pores (above 500 µm) reappear on the pores distribution chart (Figure 9f). However there is a certain difference in the distribution of pores of small size. The µCT imaging shows a full recovery of the specimen on microscopic level from the buckled state.

**Figure 10:**
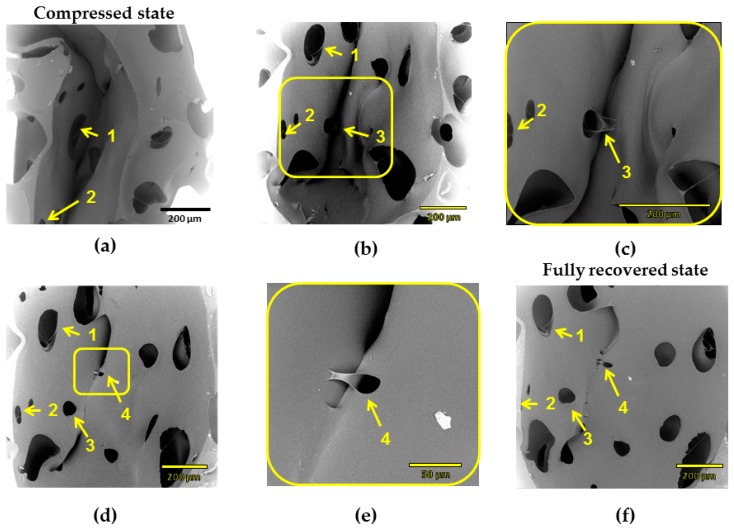
Scanning electron microscopy (SEM) images of stepwise recovery from compressed state (**a**) of intermediate density foam (cPCL-2/4/8k-180) up to the full recovered state (**f**). (**c**) Magnification of window (3) in (**b**). (**e**) Magnification of window (4) in (**d**) at an intermediate compression ratio. Yellow arrows with numbers indicate specific wall elements discussed in the text. Scale bars for images (**a**–**d**) and (**f**) are 200 µm; scale bare for image (**e**) is 50 µm.

The µCT technique has resolution limitations: elements of size below 50 µm are difficult to detect. Therefore, scanning electron microscopy (SEM) was used to study recovery process of cPCL-2/4/8k-180. SEM measurements were performed for each recovery step (Figure 10). The same cell elements are marked with yellow arrows and numbered. In densification regime (at high compression ratio, Figure 10a), cell walls are highly deformed and touch each other. At intermediate deformation ratio (Figure 10b), it is visible how a single cell started to recover its shape. However, shell of the cell and big windows stay bowed, and cell walls touch. When struts are more pronounced (like for the case amorphous low-density foam—see Supplementary Materials Figure S3), they form loops at high compression ratio.

In contrast to the amorphous foams, it was noticed that elements of different pore walls of the semicrystalline foam in densification regime form common crystalline regions. Thus, Figure 10c depicts a magnified window of a pore shell on Figure 10b. It is visible how the material from left wall is adhered to the opposite wall. The further recovery process confirms a “stickage” effect. Figure 10d depicts the recovery of element 3 and appearance of the new element (element 4). Element 4 remains partly adhered, and higher magnification also shows the “intermelted” state of cell walls, which were put to the contact during compression (Figure 10e represents magnified part of Figure 10d). In Figure 10f, a recovered state is depicted (which corresponds to 98% of recovery ratio on macroscopic scale). Some of the elements that remained interconnected are still visible, whereas a general shell-like structure of a single pore is re-established.

Such remaining interconnections of cell walls should be one of the reasons of around 2% of loss in recovery ratio (see Figure 8) for the compositions studied. Mutual crystallization/melting of walls of neighboring pores should also influence the recovery ratio and probably, programming efficiency during reversible shape-memory experiment.

#### 3.4.3. Reversible SME on Macroscale

Characterization of rSME was done for the specimens programmed by compression (compression mode) and by tension (tension mode) followed by load-free temperature cycles.

##### rSME in Compression Mode

**Figure 11:**
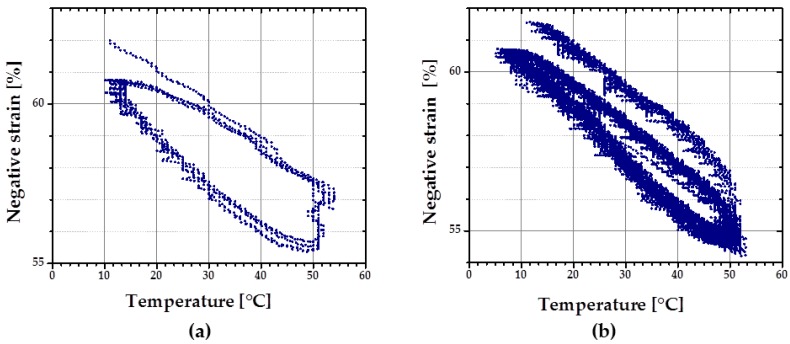
Dependence of compressive strain from the temperature change during cyclic SME tests in a load free mode of cPCL-2/4/8k-180 at heating and cooling rates of: (**a**) 3 K∙min^−1^; and (**b**) 1 K∙min^−1^.

At first, rSME experiments were performed in a compression mode. The load-free temperature cyclic test is shown in Figure 11a.

The sample expanded during heating and contracted during cooling. The maximum *ε*_rev_ achieved was 12%. This effect is similar to the behavior of PCL-based scaffold manufactured via salt casting/particulate leaching reported by Mather and co-workers [17]. This behavior is likely to be related to a combined effect of thermal expansion and reversible crystallization-melting during cyclic change of the temperature.

As the thermal expansion coefficient for polyurethane foams is relatively low (around 4 × 10^−5^ K^−1^ [24]), the observable cyclic shape change during SME tests can be rather explained by the crystallization and melting of crystallizable PCL domains. Taking into account the hindered mobility of PCL chains in the foam network, the same SME test was performed with 1 K∙min^−1^. Qualitatively, no significant difference in the reversible shape change value is observed. However, for the slow heating and cooling rate (Figure 11b), the hysteresis area between heating and cooling curves appears to be smaller.

##### rSME in Tension Mode

**Figure 12:**
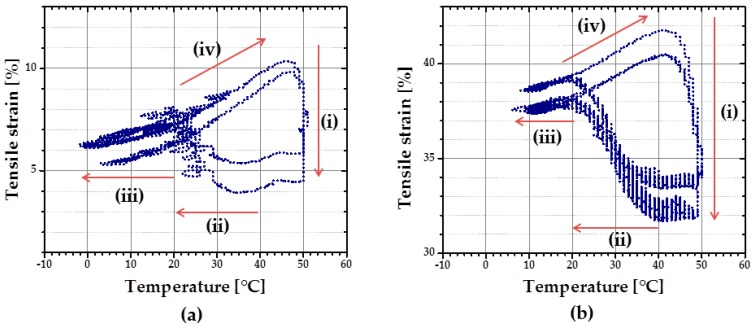
Dependence of tensile strain from the temperature change during cyclic load free SME tests of cPCL-2/4/8k-180 programmed: (**a**) parallel to foam free rise; and (**b**) perpendicular to foam free rise. Heating and cooling rates were 3 K∙min^−1^. The following four stages are marked: (i) shape recovery at *T*_high_; (ii) length increase upon cooling; (iii) contraction upon cooling; and (iv) length increase upon heating.

rSME tests in tension mode were carried out for the intermediate density foam cPCL-2/4/8k-180 (see Figure 12).

At first, the cyclic thermomechanical test was done for the specimen cut parallel to the foam rise (Figure 12a). In contrast to the compression mode there are four stages distinguishable in the tension mode during load-free temperature cycles: (i) shape recovery at *T*_high_ = 50 °C; (ii) length increase upon cooling in a range from around 40–20 °C; (iii) contraction from around 20 °C to *T*_low_ = 0 °C; and (iv) length increase during heating from 20 °C to *T*_high_ = 50 °C. Recovery at *T*_high_ = 50 °C (i) is induced by melting of crystallites (so called “entropic elasticity”) (see Figure 12a). An increase in the specimen length upon further cooling (ii) is probably related to the crystallization of the PCL-chains. Contraction upon further cooling (iii) could be related to thermal contraction. Further heating triggers the increase in specimen length (iv), which can be the complimentary effects of both thermal expansion and cold crystallization, as discussed above. The maximum *ε*_rev_ achieved for the sample cut parallel to foam rise is around 3.5%.

A similar temperature cyclic test was done with the foam specimen, cut perpendicular to the foam rise. Here, *T*_low_ was set at 13 °C to reduce low-temperature contraction effect. Two temperatures were used as *T*_high_: 45 °C during the first cycle and 49 °C for the following cycles. Specimen cut perpendicular to foam rise exhibits similar behavior as discussed above: cooling-induced crystallization and contraction, heating-induced cold crystallization and recovery (Figure 12b). However, for the specimen, cut parallel to foam rise (Figure 12a), strain increase during cooling (from *T*_high_ to *T*_low_) is almost the same as during the heating part (from *T*_low_ to *T*_high_). Whereas the contribution of strain growth during cooling (from *T*_high_ to *T*_low_) is obviously predominant for the specimen, cut perpendicular to the foam rise, compared to the heating part (Figure 12b). It seems that the reorientation of the PCL chains contrarily to their initial state, obtained during foaming process, forces the crystallization process. The maximum *ε*_rev_ achieved for the sample cut perpendicular to foam rise is around 5%.

#### 3.4.4. Reversible SME on Microscale

**Figure 13:**
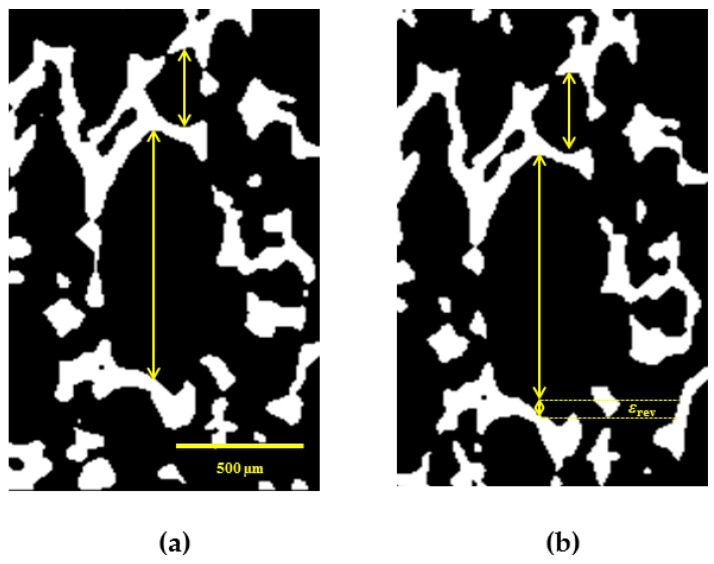
µCT image of cPCL-2/4/8k-180 in the direction of programming: (**a**) at *T*_high_ = 49 ± 1 °C; and (**b**) at *T*_low_ = 22 ± 1 °C. Yellow arrows have length of corresponding pores at *T*_high_. The gap between the arrows and the corresponding pore walls at *T*_low_ indicates an rSME of a pore (ε_rev_). Scale is 500 µm.

In this section, we present the results of investigation of rSME effect of cPCL-2/4/8k-180 on microscopic level. The sample was programmed by stretching in direction perpendicular to foam rise, as this programming direction showed the highest efficiency for the discussed foam specimens. The used custom-build sample holder of the µCT set-up did not allow cooling below 20 °C. In addition, the macroscopic rSME experiments showed the crystallization-induced elongation of cPCL-2/4/8k-180 on the temperature interval from 40 to 20 °C only (Figure 12b). For the in-situ experiments therefore the room temperature was chosen as *T*_low_. Figure 13 depicts a µCT image of a single pore with visible rSME at *T*_high_ and *T*_low_. Double sided arrows of the same length indicate the same elements at *T*_high_ and *T*_low_.

In general, the pores are longer at low temperature. For some of the pores, however, the dimensions stayed unchanged. The maximum calculated rSME was around 10% and the minimum measured rSME was around 0%. The elongation of single pores at low temperatures should be related to the crystallization-induced elongation, or to the rSME (shown as *ε*_rev_ in Figure 13b). The stability of pores’ dimensions with the temperature change (for the case of no rSME) could be attributed to the following reason: some programmed pores could only compensate the thermal contraction, which has the opposite to rSME direction, and show therefore no rSME.

It is commonly accepted that larger pores exhibit higher deformation ratio than smaller cells of the foam. Hence, better programming efficiency and higher rSME could be expected for pores of large cell size. Nonetheless, there is no clear correlation between the values of rSME and the pore size. Thus, pores of around 300 µm in the programmed state exhibited pronounced rSME of 5% to 10% similar to pores from 400 to 1000 µm in the stretched state. Another parameter that should influence the capability to rSME is the thickness of a strut of a pore. However, pores with similar width of struts exhibited rSME from 0 to 10%.

The inhomogeneous rSME effect could be related to the non-uniform, random distribution of PCL-diols during foaming and as a result to the fluctuations in density and degree of crystallinity within specimen’s volume, as well as to the different degree of stretching of pores during programming.

#### 3.4.5. Reversible SME in Bending Mode

**Figure 14:**
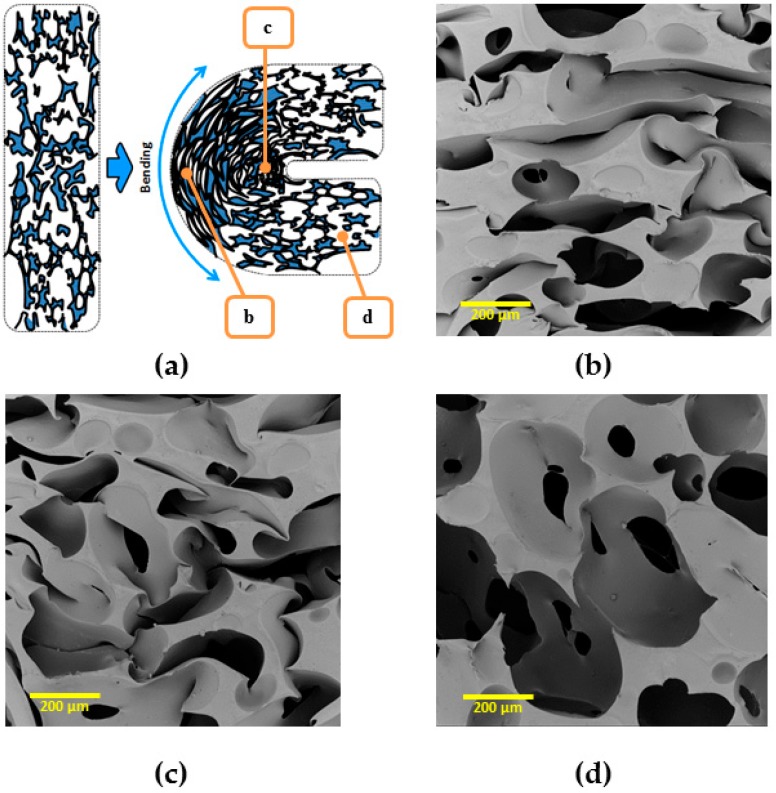
(**a**) Scheme of foam programming by bending; and (**b**–**d**) SEM pictures of the cPCL-2/4/8k-180 programmed via bending at corresponding places in bended foam specimen: (**b**) SEM image of outer side of the bend; (**c**) SEM image of innerside of the bend; and (**d**) SEM image outside the bend; scale bar is 200 µm.

To demonstrate obtained rSME, the cPCL-2/4/8k-180 was programmed by bending. The scheme of programming is shown on Figure 14a (side view). During bending, the deformation of cells of a foam specimen is irregular: foam cells appear to be elongated at the outer side of the bend (Figure 14b) and compressed at the inner side of bend (Figure 14c). The cells of foam, which are located far from the bend, are only slightly deformed (Figure 14d).

Thus, bending could be the efficient way of programming due to the combined effect of stretching-induced crystallization and interaction of walls of pores due to densification, when the additional possibility for crystallization of the material between walls is introduced.

**Figure 15:**
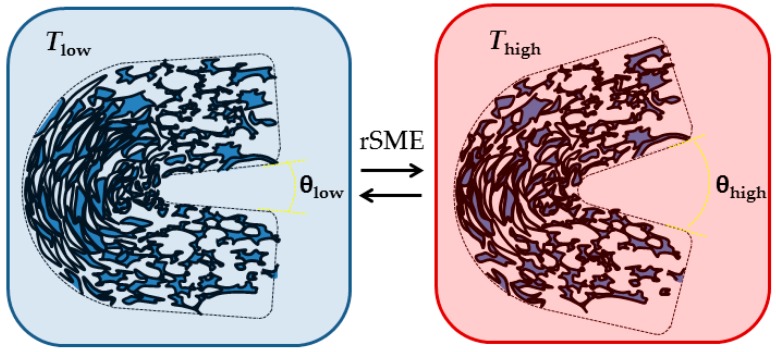
Scheme of rSME of semicrystalline water-blown foam.

At *T*_low_ therefore the pores at the outer side of the bend should elongate and the pores at the inner side of the bend should densify upon crystallization of PCL segments. This should lead to a “closing” of the structure, or a decrease in angle between edges. When *T*_high_ is reached, the pores at the outer side of the bend should contract, whereas the pores at the inner side of the bend should expand, which would lead to the increase of angle value between the edges of the specimen (see Figure 15).

**Figure 16:**
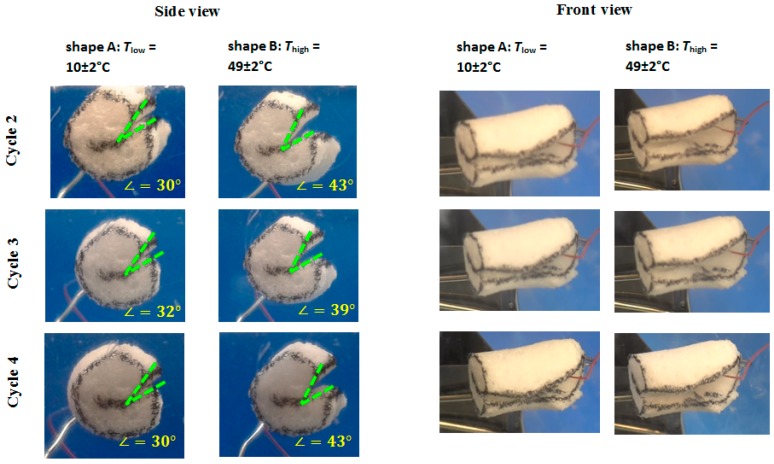
Load free temperature cycles of intermediate density foam.

Load-free temperature cycles (from 2 to 4) of an intermediate density foam sample in bending mode are shown in Figure 16. Actuation (opening of the bend to the angle θ = 42 ± 2°) was observed during heating from *T*_low_ = 10 ± 2 °C to *T*_high_ = 49 ± 2 °C, which is due to the partial melting of PCL crystalline segments. When the foam specimen was cooled from *T*_high_ = 49 ± 2 °C to *T*_low_ = 10 ± 2 °C, the bend was closing to the angle θ = 31° ± 1°. A repeatable shift in angle from shape A to shape B was ∆θ = 11° ± 3°.

## 4. Conclusions

In this study, we report the successful synthesis of a series of semicrystalline water-blown free-rise PU foams using commercially available linear poly(ε-caprolactone)-diols crosslinked with MDI. We demonstrated that rSME effect is achievable for cPCL water-blown foams, while different programming regimes yield different rSME directions and shape-recovery ratios. Surprisingly, the most pronounced rSME is achieved when the stress during programming is directed perpendicular to foam rise. The rSME was quantified with cyclic thermomechanical experiments consisting of a programming step followed by repeating heating–cooling cycles. The cyclic tests were performed in a stress-free regime in compression and tension modes. The reversible strains up to *ε*_rev_ = 12% ± 1% were achieved for the specimens tested in the compression mode, *ε*_rev_ = 3.5% ± 0.5% for the tension mode for the foam programmed in foam-rise direction, and *ε*_rev_ = 5.0% ± 0.5% was obtained in tension mode for the foam programmed perpendicular to the foam-rise direction. The switching temperatures were around 50 °C for all testing modes.

We have analyzed the SME of the foam on three different levels: the macroscopic level of the foam, the level of a single pore in the foam and thermomechanical properties of the polymeric system, where the behavior of the system on the molecular level could be studied. From the results of these investigations, it can be concluded that the foam density and the mobility of crystallizable chains determine the actuation efficiency of the crystalline domains. Another core factor for a pronounced rSME is how the deformation of an overall shape of a foam sample during programming step is translated to a single-pore level. We showed here that the mechanism of a rSME of a single pore in a tension mode is similar to the mechanism in the bulk materials (melting-induced contraction and crystallization-induced elongation). However, the programming via compression or bending of the overall sample is more complicated as it may create local up to total densifications of pores. Still, the response of a single pore in a compressed state during the rSME is an open question and should be investigated additionally.

We anticipate that our research will initiate the development of a class of smart polyurethane foams, and will introduce the corresponding features to the conventional objects, e.g., the ability to adjust their hardness and shape to the seat cushions for individual needs, or zero or negative expansion coefficient to insulation materials.

## Figures and Tables

**Table 1:** Properties of poly(ε-caprolactone) diols used for foam preparation.

Trade name	*M*_n_ (g∙mol^−1^)	OH-number ^1^ (mg·KOH∙g^−1^)	*T*_m_^2^ (°C)
CAPA 2205	2000	36	30–50
CAPA 2402	4000	32	40–60
CAPA 2803	8000	13	50–60

^1^ OH-numbers were determined by potentiometric titration on DMS-Titrino 716 (Metrohm, Filderstadt, Germany); ^2^ *T*_m_ were measured with differential scanning calorimetry (DSC) 204 calorimeter (Netzsch, Selb, Germany). *M*_n_: number average molecular weight.

**Table 2:** Compositions of water-blown PCL-based polyurethane foams. PCL: poly(ε-caprolactone); DEOA: diethanolamine.

Composition name ^1^	CAPA 2205 (wt %)	CAPA 2402 (wt %)	CAPA 2803 (wt %)	DEOA (pphp)	Water (pphp)	Dabco T9 (pphp)	Dabco BLV (pphp)	NCO-index
**cPCL-4k-220**	-	100	-	4	1	0.15	0.4	392
**cPCL-2/8k-170**	50	-	50	4	1	0.15	0.4	243
**cPCL-2/4/8k-180**	33	33	33	4	1	0.15	0.4	333
**cPCL-2/4k-130**	50	50	-	4	1	0.15	0.4	333
**cPCL-2k-110**	100	-	-	4	1	0.15	0.4	344

^1^ sample ID: cPCL-X/Y/Zk-123, cPCL denotes crosslinked PCL, X/Y/Zk stands for number average molecular weight of PCL-diol used, and the last three digits represent the density of the obtained foams.

**Table 3:** Densities and gel content values for obtained compositions. *DOC*: weight percent crystallinity; *G*: gel content; *E*: *E*-modulus.

Composition	Density *ρ* (kg∙m^−3^)	*G* (%)	*DOC* (%)	*E* at 20 °C (MPa)
cPCL-2k-110	110 ± 10	98 ± 1	-	-
cPCL-4k-220	220 ± 20	82 ± 5	23.6 ± 0.6	7 ± 2
cPCL-2/4k-130	130 ± 3	89 ± 2.3	6.0 ± 0.2	2 ± 1
cPCL-2/8k-170	170 ± 8	89 ± 6	16.8 ± 0.9	7 ± 2
cPCL-2/4/8k-180	180 ± 20	86 ± 2.5	16.5 ± 1.1	7 ± 2

## Supplementary Materials

The following are available online at www.mdpi.com/2073-4360/8/12/412/s1, Figure S1: Storage modulus dependence on the temperature for semicrystalline foams with different degree of crystallinity after extraction in DMF solvent, Figure S2: Images of the in-situ recovery process of cPCL obtained with light microscope, and Figure S3: SEM images of stepwise recovery from compressed state of a low density amorphous foam (cPCL-2k-110) up to the full recovered state. Table S1: Crosslink densities and average molecular weight between physical or chemical crosslinks obtained using Flory and Rehner approach. PCL: poly(ε-caprolactone).
