# Supplementary material for: Water-Blown Polyurethane Foams Showing a Reversible Shape-Memory Effect
Source: Polymers (Basel). 2016 Nov 28;8(12):412. doi: 10.3390/polym8120412 (PMC6431911; doi:10.3390/polym8120412)
Supplement: Supplementary file 1 [file polymers-08-00412-s001.pdf]

Supplementary Materials: Water-Blown Polyurethane Foams Showing a Reversible Shape-Memory Effect

Elena Zharinova, Matthias Heuchel, Thomas Weigel, David Gerber, Karl Kratz and Andreas Lendlein

DMTA Analysis of the Semicrystalline cPCL-PU Foams after Extraction

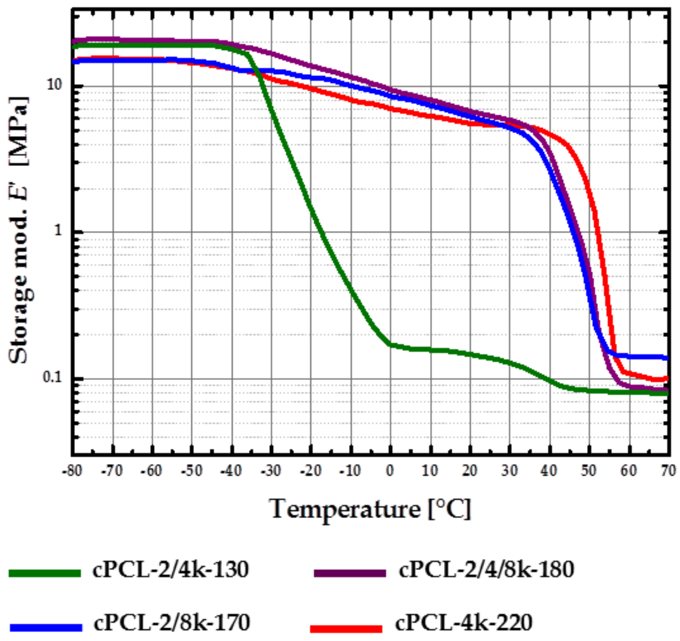

Figure S1. Storage modulus dependence on the temperature for semicrystalline foams with different degree of crystallinity after extraction in *N*,*N*-dimethylformamide (DMF) solvent. PCL: poly(ε-caprolactone).

Table S1. Crosslink densities and average molecular weight between physical or chemical crosslinks obtained using Flory and Rehner approach. PCL: poly(ε-caprolactone).

Composition	M_c [1] (g·mol^{-1})	ρ_{cross} [2] × 10^2 (mmol·cm^3)	ϕ	E' at 70 °C (Mpa)
cPCL-4k-220	16500 ± 1900	3.2 ± 0.2	0.73 ± 0.07	0.1 ± 0.01
cPCL-2/4k-130	13000 ± 1500	4.6 ± 0.3	0.81 ± 0.09	0.079 ± 0.01
cPCL-2/8k-170	9000 ± 1000	8.2 ± 0.6	0.83 ± 0.09	0.14 ± 0.01
cPCL-2/4/8k-180	15000 ± 1800	4.8 ± 0.3	0.85 ± 0.09	0.083 ± 0.01

[1] calculated using $G^* = \frac{\rho_{foam} \times RT}{M_c}$, where G* is a shear stress calculated from values of E' modulus at T = 70 °C obtained from dynamic mechanical thermal analysis (DMTA), R is the gas constant and M_c is estimated molecular weight between physical or chemical crosslinks. A Poisson ratio of 0.25 was taken for all formulations to estimate the shear stress; [2] calculated using $\rho_{cross} = \frac{2}{3} * \frac{\rho_{foam}}{(1-\phi) \times M_c}$, where ρ_{cross} stands for crosslink density and ρ_{foam} is the foam density; 2/3 is the coefficient of functionality for diethanolamine (DEOA); and ϕ is the porosity.

In-Situ Recovery of cPCL-2/4/8k-180 on a Macroscale

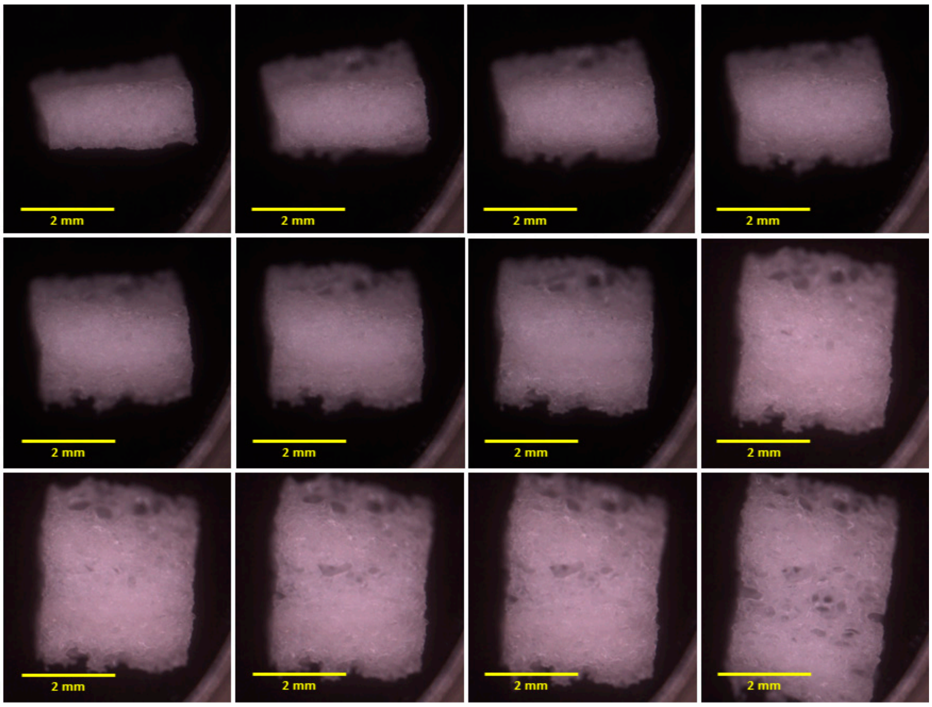

Figure S2. Images of the in-situ recovery process of cPCL-2/4/8k-180 obtained with light microscope.

In-Situ Recovery of "Amorphous" cPCL-2k-110 Foam

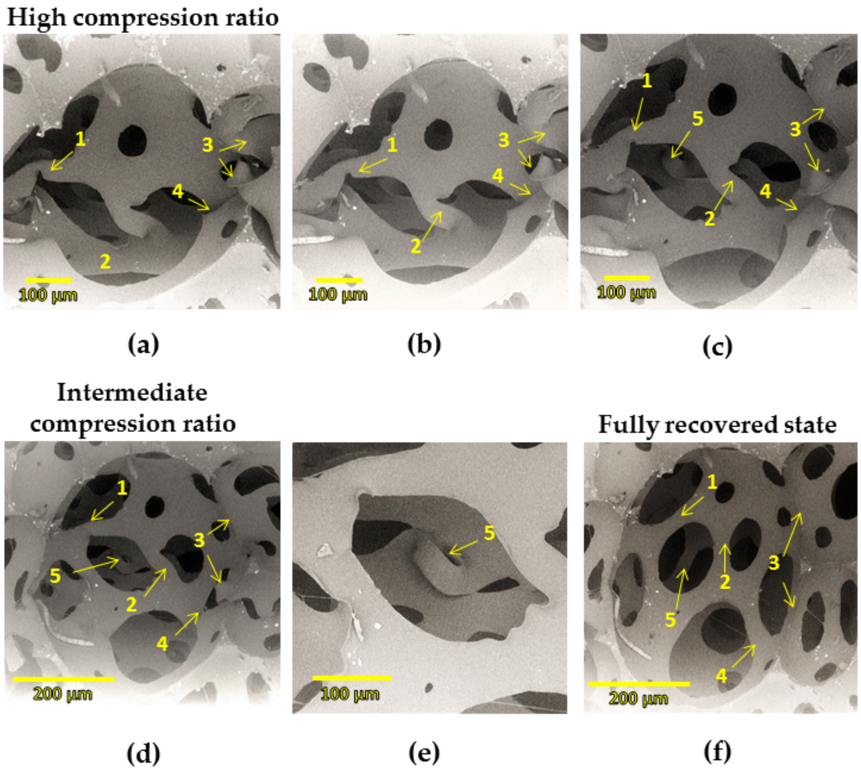

Figure S3. SEM images of stepwise recovery from compressed state (**a**) of a low density amorphous foam (cPCL-2k-110) up to the full recovered state (**f**). (**e**) A magnification of a strut loop (5) in picture (**d**) at an intermediate compression ratio. Yellow arrows with numbers indicate specific wall elements discussed in the text). Scale bare for images (**a**–**c**) and (**e**) is 100 µm, scale bare for images (**d**,**f**) is 200 µm.
